# Educational Advices for New-Graduate Nurse on Social Media in China: A Grounded Theory Study

**DOI:** 10.1155/jonm/1276010

**Published:** 2024-12-02

**Authors:** Xiaolei Liu, Xiangying Feng, Meixia Zhang, Jun Liu, Min Chen

**Affiliations:** ^1^Department of Medical Insurance, Xijing Hospital, Air Force Military Medical University, Xi'an, Shaanxi, China; ^2^Department of Gastrointestinal Surgery, Xijing Hospital, Air Force Military Medical University, Xi'an, Shaanxi, China; ^3^Department of Nursing, Xijing Hospital, Air Force Military Medical University, Xi'an, Shaanxi, China; ^4^State Key Laboratory of Cancer Biology, National Clinical Research Center for Digestive Diseases and Xijing Hospital of Digestive Diseases, Air Force Military Medical University, Xi'an, Shaanxi, China

**Keywords:** advice, education, grounded theory, new-graduate nurse, social media

## Abstract

**Aims:** This study aimed to excavate the entry advices and suggestions for new-graduate nurses.

**Background:** Research based on social media analysis for advices to new-graduate nurses is rare. We conducted a detailed analysis and modularization of the relevant contents based on social media.

**Design:** A grounded theory study based on social media content.

**Methods:** The answers to questions such like “What's your advices and suggestions for new-graduate nurses?” on a famous social media platform in China were searched, collected, coded, and analyzed until September 2023.

**Results:** Four core category modules including professional knowledge module, professional ability module, socialization and interpersonal communication module, and personal trait module were established. The theoretical framework of 21 main categories was also established.

**Conclusion:** Social media contents provide valuable and experienced advices and suggestions for new-graduate nurses in an efficient and convenient way. The analysis of these contents is helpful for new-graduate nurse education and management.

**Summary**

  What is already known about this topic?
• Social media is widely used and has a huge impact.
• Although there are still some issues, social media can play a positive role in education.
  What does this paper add?
• The advices for new-graduate nurses on social media have a high level of quality.
• These contents will supply a positive promoting effect on the role transformation and adaptation to a new environment for new-graduate nurses.
  The implication of this study:
• Education managers should pay attention to the information content on social media.
• Education managers should attach importance to the role of social media in nursing education, or as a supplement to traditional education.

## 1. Introduction

Social media is a virtual community platform that people use to create, share, and exchange opinions, viewpoints, and experiences. Social media has flourished on the tide of the Internet, and the information it spreads has become an important channel for people to obtain information. At present, social media mainly includes social networking sites, microblogs, blogs, forums, podcasts, and so on [1].

The dynamic, immediacy, diversity nature of information and rapidity of communication on social media are rapidly changing the way we participate in society [2]. In healthcare domain, social media and its data are widely used in cancer screening, public health, patient surveys, and more [3–5]. In the field of education, social media is recognized for its ability to disseminate educational messages and understand learner feedback in an efficient, concise, and interactive manner, such as using Twitter to promote sustainability in nursing education, using Facebook as an interactive learning platform for nursing students, and using Instagram to educate doctors, technicians, and nurses around the world about radiology and more [6–8].

In fact, the content information on social media is independently generated by a large number of users, so it is undeniable that the use of social media also has educational limitations and potential negative effects. Most of the content on social media is informal content posted by users spontaneously, and its information quality is uneven and lacks an objective and authoritative measurement standard. The distribution of content on social media is scattered, and it may take time and energy to obtain the required information, which also has certain requirements on users' self-learning ability and information literacy. Various kinds of information on social media also put forward certain requirements on learners' self-control ability. Learners with poor self-control may use it for entertainment purposes or may be addicted to use it, which affects normal work and study.

However, the outstanding features of social media in making up for the shortcomings of traditional education have received increasing attention [9]. Commonly used social media such as Twitter and Facebook, as well as WeChat and QQ in China, are all listed as research objects [10–12]. These studies showed the positive role of social media as well as the need for more high-quality research involving the role of different social media platforms in education.

New-graduate nurses have just entered the hospital, and they are young and full of curiosity. Social media is popular in this group. In order to adapt and integrate into clinical environment, and to realize the identity transformation from students to nurses faster and better, they usually need to overcome many difficulties and learn a lot of things. Many new-graduate nurses turn to social media for tips and advices at the beginning of their careers. However, the quality and characteristics of these advices have not been systematically reviewed and studied. At the same time, considering that informal content on open social media may be different from traditional educational content, it is necessary to study it in order to form systematic recommendations.

In this study, we try to collect and analyze the contents about the advices and suggestions for new-graduate nurses published on a well-known social media platform in China named Zhihu. The platform is a Chinese Internet high-quality question-and-answer community, taking “Let people better share knowledge, experience and insights, find their own answers” as its brand mission. With a serious, professional and friendly community atmosphere, and easily accessible quality content, the platform has gathered the most creative people in the fields of technology, education, culture, etc. and has become an influential knowledge sharing community and original content platform for creators in China. The specific question for this study was what is the content and how is the quality of the advices posted on social media for the entry of new nurses? Are these advices valuable for new nurses and nursing education? The specific goal of this study was to analyze, classify, summarize, and evaluate the overall content and quality of the advices for new nurses posted on social media. The contribution of this study to nursing education is to explore the content and quality of nursing education–related information posted on social media. The practical significance of this study is that it provides a set of methods for analyzing nursing education–related information content on social media based on grounded theory. With this method, more nursing education–related content on more social media platforms can be summarized, refined, and analyzed. Evaluating the quality of information on social media can help assess whether it can be an effective source of nursing education information and an effective supplement to nursing education, and then whether it can play a positive role in nursing education.

## 2. Methods

### 2.1. Data Sources

According to market performance, Zhihu platform is a high-quality knowledge-based social media platform in China featuring Q&A and knowledge sharing. The platform features high-quality Q&A and attracts a large number of professionals with its in-depth and original contents. Many healthcare professionals in China are active on the platform and use it for exploring problems and finding answers. In this study, the platform was selected as data source. The answers to such questions like “What are the advices or suggestions for new-graduate nurses?” are collected and analyzed. Firstly, according to the research purpose, several keywords such as “graduation,” “new-graduate nurse,” “suggestions,” and “advices” are selected. Then, use these keywords to search for relative Q&A contents on the platform published before September 2023. In this study, newly graduated nurses are selected as the research object, mainly because nurses at this stage are in the transition period between graduation and entry, and in the transition period between the roles of students and nurses. This stage is very important and often has more problems, so this group deserves more attention and more research. Although all the data in this study came from the public network and the information was published anonymously, the study was approved by the Ethics Committee because the study involved nurses in the hospital setting.

### 2.2. Inclusion and Exclusion Criteria

According to the research purpose, the inclusion criteria were formulated: the advices or suggestions for new-graduate nurses were clearly proposed. Those advices or suggestions for new-graduate nurses not explicit were excluded.

### 2.3. Data Collection

Use Excel to record the text contents of the questions and answers, including the name of question publisher, release time, content, the name of respondent, answer time, and answer content. Q&A contents are ranked according to the platform's own algorithm based on Wilson's algorithm.

### 2.4. Data Coding

**Figure 1:**
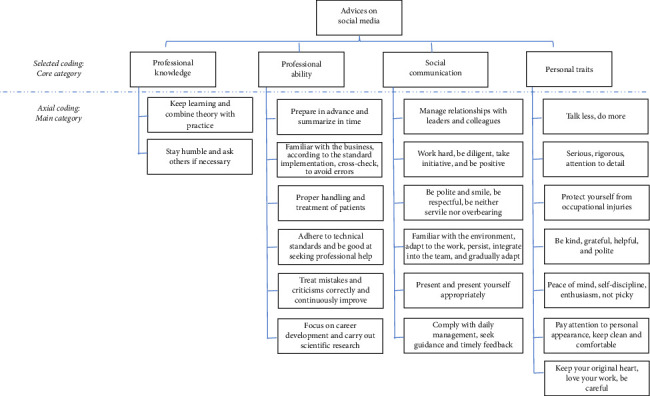
Visual representations of core themes.

The research method of grounded theory is applied in the process of data collection and analysis. Grounded theory [13, 14] was first proposed by American sociologists Barney Glaser and Anselm Strauss. It requires researchers to use their own theoretical sense to collect relevant data in the actual society without making theoretical assumptions, judge and grasp the differences and similarities and characteristics of the data, and then encode the data, summarize the specific phenomena into concepts and categories, and finally elevate them to theory. It is a bottom-up qualitative research method. Considering the complexity of Chinese characters and the characteristics of personalization and variety of network expressions, it is difficult to achieve real quantitative research, and the use of grounded theory can make up for the deficiency of quantitative analysis. Grounded theory is to define and classify data through coding in the process of data collection and assign a conceptual category, which can realize the extraction of “themes” from scattered data and help researchers establish theories closely related to the real world. The analysis steps generally include three stages: open coding, spindle coding, and selective coding. In this study, the above coding and qualitative analysis were carried out. The open coding process is mainly the integration from the original sentence to the conceptual comparison, and then to the categorization, which is the analysis and extraction of data. The main axial coding process is from the main category to the corresponding category of induction; it is the process of data reorganization and induction. The process of selective coding, from the selection of core categories to the construction of grounded theoretical models, is a process of clarifying ideas and refining. The whole process of coding is a process of continuous iterative refinement and induction from the bottom to up, from concrete to abstract, and from concept to theory. The visual presentation is shown in Figure 1.

### 2.5. Implementation Process

The two researchers independently analyzed the answer contents, read them repeatedly, and selected keywords from the contents to initially conceptualize the answer content in order to retain the original meaning of it. When an answer contains multiple layers or types of suggestions, it is marked with different identification codes according to the scope covered by it. The other two researchers read these original contents respectively, and on the basis of the above work, summarized and refined the answers and initially constructed core category modules and main categories including several aspects. In the coding process, when there is a disagreement between two researchers, the third researcher will make the judgment, and if necessary, it can be negotiated by organizing a common meeting, and it is determined according to the principle of minority obedience to the majority. For example, one suggestion mentioned that “book knowledge and clinical knowledge should be studied and mastered as much as possible and combined,” which some researchers coded as “professional knowledge” and some as “professional skills,” but after a third researcher judged that it was more appropriate to encode it as “professional knowledge,” it was finally coded as “professional knowledge.” Several researchers have medical or nursing-related professional backgrounds and many years of experience in the healthcare industry. After three stages of coding, primary categories, main categories, and core categories are formulated. See Table 1 for examples. It is important to clarify the criteria between different categories. The concepts, standards, and scope of these categories are defined as follows: professional knowledge refers to book knowledge and theories related to nursing profession; professional ability refers to the hands-on operation ability related to nursing profession and the ability to solve specific problems in practical nursing work. Interpersonal communication refers to the state pattern of getting along with others in the organization, the situation of cooperating with team members, etc., which is an external manifestation; personal traits refer to an individual's inner character, preference, habit, moral character, etc. These categories carry the contents that new nurses should pay attention to from different aspects, and their combination forms a relatively complete system.

## 3. Results

### 3.1. Number of Answers Included in the Study

505 answers were initially obtained. Fifteen answers were excluded according to the inclusion and exclusion criteria. Finally, 490 effective answers were obtained, accounting for 97% of the whole.

### 3.2. Core Category Modules and Main Categories

The study constructs a theoretical thematic framework that includes 4 core category modules and 21 main categories. The four core modules include (1) professional knowledge module, including theoretical knowledge and practical knowledge; (2) professional competence module, including professional quality, skills, and abilities; (3) socialization and interpersonal communication module, including interpersonal relationship, social environment adaptation, etc.; and (4) personal trait module, including moral character, etc.  Table 2 provides the theoretical topic framework content, and Table 3 provides the frequency of occurrence of each major category.

### 3.3. Content Analysis

#### 3.3.1. Professional Knowledge Module

There are two main categories under the core category module. “Keep learning, connect theory with practice” and “Stay humble and ask others when necessary.” This item highlights two points: First, in the attitude, to keep learning and be modest. Second, in the real world, theory needs to be combined with the actual situation, and it cannot be divorced from the reality and scripted what the book says. This item emphasizes the importance of knowledge and application.

#### 3.3.2. Professional Competencies and Skills Module

There are six main categories under the core category module. This item is refined from the perspectives of good working habits, nursing professional characteristics, nurse–patient relationship, nursing technical specifications, continuous improvement, and nursing scientific research, highlighting the importance of nursing professional ability and accomplishment.

#### 3.3.3. Socialization and Interpersonal Communication Module

There are 6 main categories under this core category module. This item is refined from the perspectives of interpersonal relationship, work attitude, environmental adaptation, self-display, compliance with rules, and discipline, highlighting the importance of interpersonal relationship and social adjustment.

#### 3.3.4. Personal Traits Module

There are 7 main categories under this core category module. The item is mainly considered from the perspective of self-management and connotation literacy, including many aspects of the quality or style of being a person and doing things. Some of the contents seem to have a conceptual overlap with some items previous on socialization and interpersonal communication, but their emphasis is different. The former highlights interpersonal and mutual communication, but this item more highlights personal cultivation.

## 4. Discussion

### 4.1. Overall Information Quality

This study explored the contents of advices and suggestions for new-graduate nurses on a well-known social media platform in China. It showed how social media can provide a channel for people to share their experiences and knowledge equally. Contents varied in subjects and styles, from concise to verbose, macro to micro, and included information on nursing expertise, skills, socialization, and personal characteristics. Most of the advices were positive and valuable. However, it must be noted that information on social media, without formal peer review, is posted by users themselves. Media platforms generally regulate whether information involves clearly illegal content, but do not interfere too much with the quality of the information itself. As a result, it is often assumed that the quality of information on social media will vary. In this study, the overall quality of the information content obtained may be high due to the high quality of the selected platform itself, but there are also individual advices that are too simple or not highly relevant. In addition, some content has a certain tendency or uses Internet or spoken language, which may mislead viewers and cause difficulty in understanding.

### 4.2. Theoretical Subject Framework

In the field of evaluating the quality of new-graduate nurses, some researchers have established relevant systems [15–17], most of which include professional knowledge, professional ability, quality cultivation, personal characteristics, and other aspects. In this study, the theoretical theme framework extracted from the advices and suggestions on social media basically covered the above aspects. These showed that social media, as a kind of informal mass media, had high quality in its contents. Social media can be used as one of the channels for caregivers to obtain high-quality knowledge and information.

### 4.3. Professional Knowledge

Nursing work is highly professional and requires a solid foundation theoretical knowledge. At the same time, nursing work is also highly practical and requires an effective combination of theory and practical knowledge. As we all know, only in practice can we deepen the understanding and grasp of theoretical knowledge. At this point, maintaining an attitude and approach of humble learning becomes particularly important for new-graduate nurses, and many advices and suggestions mention this aspect.

Some of these responses are often rare in public textbooks and learning materials, which may involve the category of “tacit knowledge” [2]. Practical experiences tell us that “tacit knowledge” is crucial for new-graduate nurses to enhance their abilities.

### 4.4. Professional Competence

New-graduate nurses need to possess multiple abilities, including planning, summarization, adaptability, problem-solving skills according to regulations, and so on. These abilities contribute to a good career start. New-graduate nurses also need to have the ability to be rigorous and meticulous, avoid errors, and handle doctor–patient relationship, which are almost essential skills for this job. When encountering difficulties, seek advices from colleagues in a timely manner without feeling embarrassed. When mistakes occur, do not intentionally conceal them out of fear. Humbly accept criticism from colleagues and superiors. In summary, as a new employee in the professional field, new-graduate nurses need to constantly enhance their professional abilities, master skills, improve professional level, and solve difficulties and problems, in order to provide high-quality services for patients and also have a good career development for themselves.

### 4.5. Interpersonal Communication and Socialization

As we all know, professional factors only account for a part for career development, while nonprofessional factors such as interpersonal relationship often account for a considerable proportion. The characteristics of teamwork and providing quality services in nursing industry determine that; nurses especially new-graduate nurses need to pay special attention to interpersonal communication. Generally speaking, new-graduate nurses need to adapt to the department environment, integrate into the team of colleagues, showcase themselves appropriately in their work, get along well with others, be neither humble nor arrogant, and handle their relationships with patients properly. Finally, it is mentioned in many answers collected in this study that as a new-graduate comer to the workplace, they need to strictly abide by the daily management regulations of the department, and timely request and report to their superiors when they encounter important problems. This is different from the student age, but very important. The socialization process of the profession helps facilitate the transition of new-graduate nurses from graduates to qualified nurses [18]. However, as some educational scholars have pointed out, the socialization process is a complex one involving many influencing factors [19]. This process is often accompanied by some kind of painful experience. How to accelerate the process of socialization has always been a difficult challenge and a complex and sensitive topic [20, 21]. In the formal teaching environment, it is often difficult to obtain effective social information. However, on social media, the restrictions of identity, position, and rank are broken down, and people can communicate and interact freely. In a relaxed atmosphere, various messages on this issue are passed on informally [22]. This suggests that social media is a potentially powerful tool for accelerating the social process for new-graduate comers.

### 4.6. Personal Trait

Everyone's traits may be different, but there are some recognized good traits, such as diligence, steadfastness, seriousness, sincerity, good emotional management, and good personal image, which have been mentioned on social media in this study. In addition, many of the answers kindly remind new-graduate comers to pay attention to protecting themselves and avoiding occupational injuries. Similar findings can also be seen in some studies [23, 24].

Training qualified new-graduate nurses has always been a problem of great concern in the nursing field. Deficiencies in the abilities of new-graduate nurses include advanced skills, critical thinking, communication skills, and teamwork, most of which are related to “soft” skills. This suggests that new-graduate nurses need to master not only “hard” nursing skills in the cognitive areas but also soft skills in the emotional areas [25]. Encouragingly, these issues are addressed in the advices collected in this study. In light of the above suggestions from social media, managers need to proactively provide a variety of support and guidance to new-graduate nurses [26], including training programs, supportive instructors, and department culture that promotes learning and encourages new-graduate nurses to ask questions and seek feedback without fear of criticism or rudeness [27].

On the two categories of “socialization and interpersonal communication” and “personal traits,” here are the clarifications: In fact, these two categories seem to be conceptually related, but they are different in connotation. Socialization focuses more on the state, characteristics, and qualities shown by new nurses in their interactions with the surrounding people and environment, which indicates whether new nurses can better adapt to the society and environment. The personal traits of new nurses are more focused on their internal personality, emotions, habits, preferences, tendencies, and other aspects. Some of these excellent qualities are innate or gradually possessed in the process of growth, and some can be restrained or strengthened through acquired education, with inherent characteristics. Between the two, although some expressions seem to overlap in content, the connotation includes different directions and angles. Both aspects are mentioned a lot in the advices obtained in this study, so this study will take them as different independent categories.

### 4.7. Comparisons With Other Studies

Rashid, McKechnie, and Gill [22] studied advices for new doctors on the social media site Twitter. The overall design of their study is similar to our study, but there are some differences in methods and results. In terms of data acquisition, Symplur, a social media analysis software program, was used in their study to extract data, while our study was conducted manually. The way of using software is more efficient but may be limited by specific platforms or languages, while the way of manual retrieval is less efficient but may be more applicable. In terms of data analysis, their study uses a method similar to grounded theory, but the authors do not explicitly explain it. At the same time, the introduction to the information coding process is also relatively simple, while the description in our study is more specific, which is more convenient for other researchers to refer to. In terms of research results, there are also some differences. For example, their study found that “sense of humor” plays an important role in the socialization process of new doctors, while in our study, there are few suggestions of “the sense of humor.” This difference in results can be understandable and explicable given the differences between Eastern and Western cultures, organizations, and individual characteristics of the crowd. Another study used software tools to collect and analyze online contents. O'Connor and colleagues [28] conducted a descriptive exploratory study to examine the future of nursing in an age of multimorbidity. They organized a long time Twitter chat to discuss this important area. Their communication contents online were collected and analyzed by using the software tool NVivo and Keyhole. When the amount of data is large, manual extraction and analysis may be cumbersome. At this time, it may be a better choice to introduce software tools for data extraction and analysis. But in many cases, using such software still requires some manual proofreading work.

### 4.8. Limitations

The limitation of this study is that only one social media was selected, which may cause bias in selection. Collecting data from a single platform is a limitation of this study, and future research will consider expanding the scope of social media platforms. However, the social media platform selected in this study also has its outstanding characteristics, that is, it is a knowledge-based, professional social media platform. At present, other mainstream social media platforms are mostly life, entertainment, or comprehensive platforms. On these platforms, there may be questions similar to “What is the advice for new nurses” mentioned in this study, but the number may not be as large as the selected platform, and the distribution may not be as concentrated as the selected platform. For example, Weibo, another well-known social media platform in China, is a comprehensive platform. We tried to search the content of advice for new nurses on the platform and only found two related advices. However, as a possible direction for the next research and an attempt, future studies will consider including more types of social media platforms.

## 5. Conclusion

The study found that the contents related to “advices and suggestions for new-graduate nurses” on social media platforms not only involve professional knowledge and skills but also involve interpersonal communication and personal characteristics. These contents are of high quality and can provide valuable guidance for young nurses, making an efficient and convenient source of information for them. The analysis of social media contents helps nursing educators understand the cognitive knowledge gap of new-graduate nurses and to take supportive countermeasures to better integrate new-graduate nurses into clinical work. In addition, the application of the grounded theory to the analysis of nursing education contents on social media has also been verified as feasible, with the characteristics of simple operation and clear steps. The academic significance of the study is that through the method of grounded theory, the quality of nursing education information on social media is studied, the feasibility of using grounded theory method in such research is explored, and the positive role of social media in nursing education is also discussed. This can enlighten nursing educators to expand their thinking and vision and to carry out similar studies based on more social media platforms and provide methodological support. The practical significance of this study is that social media, especially high-quality social media, can be used as an effective source of information and auxiliary means for nursing education, and the method based on grounded theory is also an effective method to analyze the information on social media. This can inspire nursing educators and managers to pay more attention to educational information on social media in addition to traditional teaching methods, explore the important and valuable information in the method of grounded theory, and apply it to the practice of nursing education. At last, young nurses should be guided by the educators to use social media correctly and try to use social media with strong professional and good reputation. Ultimately, it will benefit many parties.

## Figures and Tables

**Table 1:** Examples of coding process.

Original text	Conceptualization	Primary category	Main category	Core category
“The more expertise you have, the fewer mistakes you'll make. Of course, it is clinical knowledge, not just textbook knowledge, that links theory to practice”	Professional knowledge, theoretical, practical, clinical knowledge, textbook knowledge	Knowledge theory correlation	Keep learning. Combine theory with practice	Professional knowledge
“Attach importance to scientific research papers and patents. There is no shortage of nurses who can work, but nurses who can both work and do science are scarce”	Scientific research, papers, patents, scientific research	Competence correlation	Focus on career development and carry out scientific research	Professional ability and skill
“In a new environment, sometimes relationships in the department are not as simple as you think. It's okay to have problems between nurses and patients, but it's really hard to get along with colleague.”	New environment, department, interpersonal relationship, conflict, colleagues, get along, difficult	Interpersonal relationship	Manage relationships with leaders and colleagues	Socialization and interpersonal communication
“If you're really good, show it”	Really, great, show	Display and display	Behave and present yourself appropriately	Personal traits and personal considerations

**Table 2:** Core category module and main category theory theme framework content.

Content source	Core category	Main category
Social media platforms included in the study	1. Professional knowledge module	1.1. Keep learning and combine theory with practice
		1.2. Stay humble and ask others if necessary
	2. Professional competence and skills module	2.1. Prepare in advance and summarize in time
		2.2. Be familiar with the business, carry out according to the standard, check repeatedly to avoid mistakes
		2.3. Proper handling and treatment of patients
		2.4. Adhere to technical standards and be good at seeking professional help
		2.5. Treat mistakes and criticisms correctly and continuously improve
		2.6. Focus on career development and carry out scientific research
	3. Socialization and interpersonal communication module	3.1. Manage relationships with leaders and colleagues
		3.2. Work hard, be diligent, take initiative, and be positive
		3.3. Be polite and smile, be respectful, be neither servile nor overbearing
		3.4. Get familiar with the environment, integrate into the team, persevere, and gradually adapt to the work
		3.5. Present and present yourself appropriately
		3.6. Comply with daily management, seek guidance and timely feedback
	4. Personal traits and personal considerations module	4.1. Talk less, do more
		4.2. Serious, rigorous, attention to detail
		4.3. Protect yourself from occupational injuries
		4.4. Be kind, grateful, helpful, and polite
		4.5. Peace of mind, self-discipline, enthusiasm, not picky
		4.6. Pay attention to personal appearance, keep clean and comfortable
		4.7. Keep your original heart, love your work, be careful

**Table 3:** Main categories, frequency, and content examples.

Core category	Main category	Frequency	Example
Professional knowledge module	Keep learning. Combine theory with practice	30	“The more expertise you have, the fewer mistakes you'll make. Of course, it is clinical knowledge, not just textbook knowledge, that links theory to practice”
	Stay humble and ask others if necessary	86	“At the beginning of your career, you should learn and see a lot. Ask if you don't understand. Teachers will teach you a lot if you work hard”

Professional competence and skills module	Prepare in advance and summarize in time	6	“No matter which department you go to, familiarize yourself with the special features of the department in advance and cherish the time you spend in the operating room, which may be a place you only visit once in your career”
	Familiar with the business, according to the standard implementation, cross-check, to avoid errors	63	“On closer inspection, medical errors are obvious errors”
	Proper handling and treatment of patients	9	“Especially for nurses, you're dealing not only with colleagues and superiors, but also with patients. The first impression you give a patient may determine how much they trust you”
	Adhere to technical standards and be good at seeking professional help	3	“You have to know what to do and what not to do. You must not only stick together, but also learn to say no”
	Treat mistakes and criticisms correctly and continuously improve	13	“Criticism from teachers or older staff, or directing your work, may not be understood at the time. But in fact, most of the teachers and old staff are protecting us. Therefore, we should accept criticism and guidance with an open mind. They are based on experience to avoid mistakes”
	Focus on career development and carry out scientific research	9	“Attach importance to scientific research papers and patents. There is no shortage of nurses who can work, but nurses who can both work and do science are scarce”

Socialization and interpersonal communication module	Manage relationships with leaders and colleagues	46	“In a new environment, sometimes relationships in the department are not as simple as you think. It's okay to have problems between nurses and patients, but it's really hard to get along with colleagues”
	Hard working, diligent, proactive, positive	52	“In fact, diligence is most needed for nursing work, especially for nurses who have just graduated”
	Be polite and smile, be respectful, be neither servile nor overbearing	27	“Be polite, share with others, say hello to the teacher”
	Familiar with the environment, adapt to the work, persist, integrate into the team, gradually adapt	13	“It's important to adapt!”
	Behave and present yourself appropriately	6	“If you're really good, show it”
	Follow the daily management, seek guidance, timely feedback	9	“Don't be late and don't leave early”

Personal traits and personal considerations module	Less talk and more action	31	“Less talk and more work. If you don't know what to do, ask an older person”
	Serious, rigorous, attention to detail	41	“Work on the details. Sometimes even if the details are done well, there can still be mistakes. So don't cut corners at work, just do what you can. A nurse's job is to be strict”
	Protect yourself from harm	10	“Protect yourself and don't clash with the patient's family”
	Be kind, grateful, helpful, and polite	10	“Thank those who helped you!”
	Peace of mind, self-discipline, warm-hearted, not critical	10	“It's normal to have a lot of stress. You should learn to regulate stress, regulate emotions, and release yourself! Attitude matters! If you change your attitude, it will be easier for you!”
	Pay attention to personal appearance, keep clean and comfortable	4	“Watch your image. If you make a good first impression, people will be tolerant of you. If you make a bad first impression, you may be stereotyped later in your career”
	Keep your original heart, love your work, be careful	12	“In any case, you should hold yourself to your own standards. Nursing is a work of conscience”

## Data Availability

The original data presented in the study are included in the article and tables; further inquiries can be directed to the corresponding author.
